# Den Use Patterns of Endangered San Joaquin Kit Foxes in Urban Environments May Facilitate Disease Transmission

**DOI:** 10.3390/ani15020239

**Published:** 2025-01-16

**Authors:** Brian L. Cypher, Alyse Gabaldon, Erica C. Kelly, Tory L. Westall, Nicole A. Deatherage

**Affiliations:** Endangered Species Recovery Program, California State University-Stanislaus, 1 University Circle, Turlock, CA 95382, USA; alysegabaldon@yahoo.com (A.G.); ekelly@esrp.csustan.edu (E.C.K.); twestall@esrp.csustan.edu (T.L.W.); nic.deatherage@gmail.com (N.A.D.)

**Keywords:** den use, disease transmission, endangered species, San Joaquin kit fox, sarcoptic mange, urban environment, *Vulpes macrotis mutica*

## Abstract

A robust population of endangered San Joaquin kit foxes (*Vulpes macrotis mutica*; SJKFs) occurs in the city of Bakersfield, CA. In 2013, sarcoptic mange was detected and significantly reduced SJKF abundance. Dens may be a mode of mange mite transmission. Kit foxes use dens daily and sometimes share dens. Also, mange mites are able to live off-host in den soil for multiple days. We monitored den use patterns of 37 marked kit foxes. Radio-collared foxes were tracked to dens and then those dens were monitored with cameras for 7-day sessions, the period of time mites might persist in the dens. Other foxes used the same den as a collared fox during 89.0% of sessions and the mean number was 2.5 foxes. An average of 1.8 foxes used a den concurrently with the collared fox. During 120-day intervals, the minimum survival time for kit foxes contracting mange, collared foxes used a mean of 7.6 dens, 9.8 other foxes used the same dens, and 7.3 foxes used the dens concurrently with the collared foxes. Thus, the potential for kit foxes to transmit mange through den sharing in the urban environment is considerable and may explain the rapid spread of mange throughout this population.

## Simple Summary

Sarcoptic mange, caused by skin mites, threatens a population of endangered San Joaquin kit foxes in Bakersfield, CA. The disease is always fatal to kit foxes. Kit foxes use earthen dens daily and the mites dropping off foxes may persist in the soil of dens for up to 7 days. By monitoring radio-collared foxes, we found that other foxes frequently used the same den within 7 days after use by a collared fox. Not uncommonly, foxes were in the same den at the same time, thereby enhancing the potential for mites to spread from one fox to another. In the time that it takes a fox to die of mange, it can contaminate multiple dens with mites. Den sharing is particularly common in urban environments due to the high density of foxes and extensive overlap in home ranges. This may explain the rapid spread of mange throughout this kit fox population and a subsequent population decline.

## 1. Introduction

The San Joaquin kit fox (*Vulpes macrotis mutica*; SJKF) historically ranged throughout arid shrub and grassland habitats in the San Joaquin Valley of central California. Widespread agricultural, industrial, and urban development over the past 100 years has resulted in extensive habitat destruction and extirpation of SJKFs throughout much of their range [1,2]. Consequently, the SJKF was listed as Threatened in California and as Endangered in the United States. Remaining foxes likely number less than 5000 and persist in a metapopulation consisting of three main populations and less than a dozen smaller satellite populations [2,3,4]. Historically, disease had not been identified as a significant threat to the SJKF [1].

One of the largest remaining populations of SJKFs occurs in the city of Bakersfield. This population is important for the conservation and recovery of this species as it serves as a hedge against catastrophic events in natural lands, enhances genetic diversity, and can serve as a source population for reintroductions [5,6]. Until recently, this population appeared to be stable and may have even been expanding, whereas most other SJKF populations are declining due to continuing habitat loss [3]. However, in March 2013, sarcoptic mange was detected among kit foxes in Bakersfield, and many of the cases were fatal [7]. Sarcoptic mange is caused by a mite, *Sarcoptes scabiei,* that burrows into the epidermal layers of the skin, causing intense pruritus (itching) and dermatitis as well as alopecia (hair loss), hyperkeratosis (skin thickening), encrustations, and secondary bacterial infections which may result in extreme morbidity and death. To date, no evidence indicates that kit foxes are able to recover from mange without medical treatment, and infected animals typically die within 3–5 mo [7]. *Sarcoptes scabiei* can infest various species including coyotes (*Canis latrans*), red foxes (*V. vulpes*), and domestic dogs (*Canis lupus familiaris*) [8], and mange in kit foxes likely resulted from a “spillover” event from one of these species [9]. Among red foxes, outbreaks of mange have caused catastrophic population declines of 50–98%, and some of those populations have not subsequently recovered [10,11]. Such severe population reductions or local extirpation could significantly imperil the SJKF.

After the first case detection in 2013, the disease spread rapidly throughout the Bakersfield SJKF population [7]. As of August 2023, at least 454 cases of mange in SJKFs had been documented, including at least 90 confirmed deaths. These numbers represent just a fraction of the animals contracting mange and dying, as many cases and fatalities went undetected. Evidence to date indicates that kit foxes are unable to recover from mange without treatment, and they do not appear to develop any immunity to the disease [7]. In the absence of any mitigation efforts, a significant population decline is likely. Indeed, from 2015 to 2019, SJKF detection rates at camera stations distributed across Bakersfield declined from 64.8% to 20.9% [12], indicating a marked reduction in kit fox abundance. In early 2019, a kit fox with mange was detected in the town of Taft located approximately 50 km west of Bakersfield. The origin of mange in Taft foxes is unknown. Since the first detection, at least 56 cases of mange in SJKFs in Taft have been documented, including seven fatalities. As with the Bakersfield population, these are undoubtedly underestimates of the actual cases and fatalities.

The process by which mange is transmitted between individual foxes is uncertain. Generally, contact between individuals is necessary for transmission of the mites that cause mange. However, physical contact commonly occurs among individuals in a social unit but rarely occurs between individuals of different social groups [13]. Despite this, mange spread rapidly throughout both the Bakersfield and Taft kit fox populations.

Kit foxes are obligate den users and use a den every day of the year [14]. In an earlier effort to model mange transmission dynamics, Montecino-Latorre et al. [15] concluded that den sharing among kit foxes was likely a significant factor in the intraspecific transmission of mange among kit foxes. In that study, it was assumed that mite transmission occurred by foxes coming into contact within the dens. However, Arlian et al. [16] determined that mange mites could survive for some period of time off-host if conditions (particularly temperature and humidity) were appropriate. Loredo et al. [17] measured climatic conditions within kit fox dens in Bakersfield and then applied criteria from Arlian et al. [16] to the results to estimate how long mites might be able to survive off-host in the dens. They estimated that mites might be able to survive in the soil of the dens for a mean time of 2.0 days in summer, 7.4 days in winter, and 4.8 days overall. These results indicate that if a kit fox with mange uses a den, then another fox can potentially become infested with mange mites by using the same den even if the den is not used simultaneously by the two foxes. Thus, the use of common dens could be a mode of mite transmission that facilitates the rapid spread of mange among kit foxes, particularly in urban environments where survival rates, reproductive rates, and, consequently, densities are relatively high [5,18], resulting in greater use of common dens. Sharing of dens, burrows, or resting sites is suspected in the transmission of mange among bare-nosed wombats (*Vombatus ursinus*) [19] as well as a suite of sympatric carnivores inhabiting the Białowieza Forest in Poland [20].

We investigated den use patterns by urban SJKFs in Bakersfield, CA to assess the potential for den use to facilitate intra-specific spread of disease, particularly sarcoptic mange. Our objectives were to determine (1) the number of additional foxes that could potentially become infested with mites by using a den that was used by a fox with mange and (2) the number of dens that a given fox with mange could potentially contaminate before it died from mange.

## 2. Materials and Methods

### 2.1. Study Area

**Figure 1:**
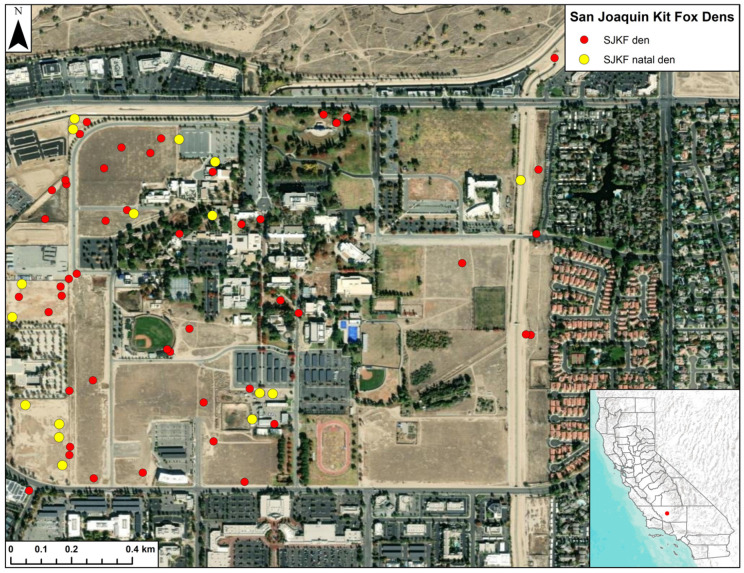
Campus of the California State University, Bakersfield in Bakersfield, CA and locations of dens used by radio-collared San Joaquin kit foxes between June 2022 and April 2023.

This project was conducted on the California State University, Bakersfield (CSUB) campus in Bakersfield, CA (Figure 1). The campus is approximately 152 ha (375 ac) in size. It is surrounded by urban land uses consisting primarily of commercial and residential developments. Irrigated lawns and landscaping are present around buildings and on athletic fields. However, large portions of the campus are unirrigated and covered by dense growth of ruderal plants, particularly non-native species such as red brome (*Bromus madritensis*), wild barley (*Hordeum murinum*), black mustard (*Brassica nigra*), and puncture vine (*Tribulus terrestris*).

SJKFs are abundant on the campus and in 2022–2023, when data were collected for this study, approximately 3–4 dozen animals were estimated to be using parts or all of the campus, based on camera station monitoring conducted by our group and the Hall Wildlife Lab. Kit foxes with mange have been detected relatively regularly on the campus since the beginning of the epidemic in Bakersfield, with 152 cases documented since 2013. Also, numerous kit fox dens had been located on the campus during previous projects [17,21].

### 2.2. Kit Fox Live-Trapping and Radio-Collaring

**Figure 2:**
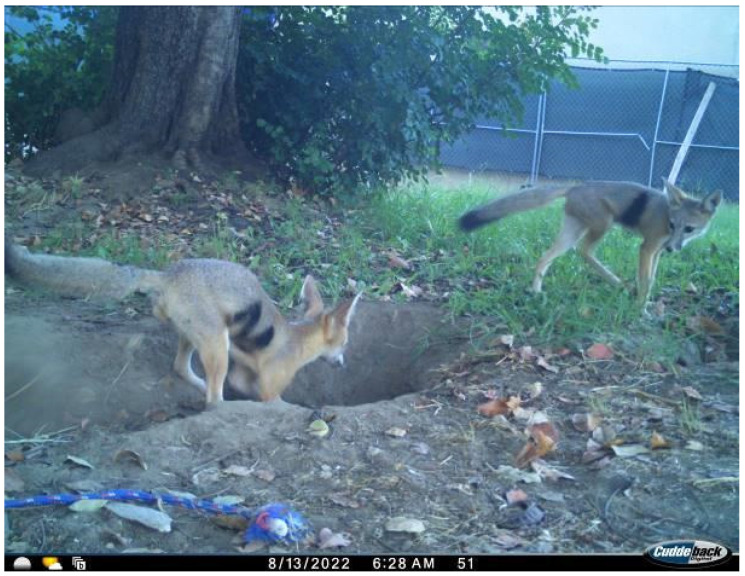
Two kit foxes with dye marks on the California State University, Bakersfield campus, August 2022.

Live-trapping for kit foxes was initiated in June 2022. Additional trapping efforts were conducted in winter 2022–2023 to collar additional foxes, particularly young-of-the-year foxes that previously were not of sufficient weight to wear a radio-collar. Kit foxes were captured using wire-mesh live-traps (38 × 38 × 107 cm; Tomahawk Live Trap, Hazelhurst, WI, USA) baited with a protein item (e.g., hot dogs, canned cat food, hardboiled eggs) and covered with tarps to provide protection from inclement weather, sun, and irrigation sprinklers. Traps were set in the late afternoon or early evening and then checked beginning around sunrise the next morning. Captured kit foxes were coaxed from the trap into a denim bag and handled without chemical restraint. Data collected for each fox included date, location, sex, age (adult or juvenile, based on tooth wear and body mass), mass, and dental condition, and a uniquely numbered tag was placed in one ear. Also, a non-toxic permanent hair dye (Nyanzol-D; Albinal Dyestuff, Inc., Jersey City, NJ, USA) was used to create a unique symbol on both sides of each fox so that it could be identified in images collected by field cameras (Figure 2). All foxes, regardless of whether they exhibited signs of mange, were also topically treated with the acaricide selamectin (Revolution; Zoetis, Florham Park, NJ, USA) at a dosage of 6.0 mg/kg body mass. To prevent the transfer of mange between captured foxes, handlers wore nitrile gloves that were discarded after handling a fox and a clean handling bag was used for each fox.

Foxes that were sufficiently large (i.e., females > 2 kg, males > 2.4 kg) were fitted with collars (Quantum 4000E Micro Mini Collars; Telemetry Solutions, Concord, CA, USA) equipped with a GPS tracking unit and a VHF transmitter with a mortality sensor. The GPS units were programmed to collect four locations per night at varied times each night. Each unit included a UHF download function so that data could be downloaded remotely using a base station (4000ER Base Station; Telemetry Solutions, Concord, CA, USA). All foxes were released at the capture site.

### 2.3. Kit Fox and Den Monitoring

Once each week, we attempted to locate the VHF signal of each radio-collared fox using a telemetry receiver (Model R1000; Communications Specialists, Inc., Orange, CA, USA). Telemetry signals were initially detected using an omni-directional antenna (Model RA-5A; Telonics, Mesa, AZ, USA) magnetically mounted on the roof of a vehicle. Once a signal was detected, a 3-element handheld Yagi antenna (Model RA-150; Communications Specialists, Inc., Orange, CA, USA) was used to navigate to the location of a given fox, which was typically a den. Each new den was assigned a unique number, and its coordinates were recorded on a cell phone using the AmigoCloud application (AmigoCloud, Seattle, WA, USA). We also attempted to download location data from the collars each week.

The use of each den by kit foxes was monitored using an automated camera station. We used Cuddeback Digital Black Flash IR cameras that employ a “black flash” infrared LED flash—that creates almost no light visible to humans—and also take high-resolution images. The black flash causes less disturbance to animals, and the lack of a bright flash significantly reduces the potential to alert people to the presence and location of the camera and therefore reduces the potential for vandalism or theft of the camera station or disturbance to the den. The cameras were programmed to take three photographs with each trigger. The trigger speed was <1 s, the sensitivity was set to moderate, and the resolution was set to high (20 megapixels). The camera stations were operated at each den for seven nights, which was the mean maximum estimated time that mange mites might survive off-host in a den [17].

### 2.4. Data Summary and Analysis

At the end of each week-long monitoring session at a given den, images were downloaded from each camera and reviewed. By counting the number of other foxes that visited a given den during the monitoring session, we estimated the number of additional foxes that might become infested with mites if the fox originally tracked to the den had mange. The number of individual foxes using the den was determined for the first two nights, first four nights, and, finally, all seven nights that the camera was operated. The number of nights corresponded to the mean estimated time that mites might survive off-host in the den in summer, across all seasons, and in winter, respectively [17]. We also estimated the number of foxes that were known to be in the den concurrently with the original fox tracked to that den. This provided an estimate of the number of foxes that could have potentially been infested with mites through direct contact if the original fox had mange.

Seasons were defined as summer (June–September), fall (October–November), winter (December–January), and spring (February–April). The summer months corresponded to the pup dispersal period, fall corresponded to the pairing period, winter corresponded to the breeding period, and spring corresponded to the pup rearing period.

For the two-day, four-day, and seven-day intervals, we determined the frequency of monitoring sessions in which other foxes were detected using a den to which a radio-collared fox had been tracked. We used contingency table analysis and a Pearson chi-square test to compare frequencies among seasons and between sexes. For each of the time intervals, we used the General Linear Models function in SPSS (SPSS Statistics package, ver. 29.0.1.1; IBM, Armonk, New York, NY, USA) to conduct a two-way analysis of variance to compare the mean number of other foxes detected using a den to which a radio-collared fox had been tracked as well as the mean number of foxes that were known to be in the den concurrently with the radio-collared fox. For these analyses, the model included season and sex as fixed factors and a sex*season interaction term. Means were compared among seasons using a Least Significant Difference multiple comparison test. For the spring season, we determined which of the radio-collared foxes were associated with a litter of pups either as the mother, father, or a helper. For each of the three time intervals, we then compared the mean number of other foxes detected using a den to which a radio-collared fox had been tracked using *t*-tests.

We estimated the number of dens that a fox with mange might contaminate with mites before it died. Kit foxes that contract mange typically die within 4–5 months if they are not treated [7]. We conservatively used 120 days as the period between disease onset and death. For each radio-collared fox that was monitored for at least 120 days, we determined the number of times that the fox was detected by radio-telemetry or camera station, the number of unique dens used by that fox, the number of other kit foxes using a den to which the radio-collared kit fox had been tracked, and the number of other kit foxes that were detected using a den concurrently with the radio-collared fox. These numbers were tallied for each fox for 120 days, beginning at the time it was first tracked to a den. A two-way analysis of variance was used to compare the mean numbers among seasons and between sexes and to identify any interactions between these two variables.

To examine spatial overlap among the radio-collared foxes, we used the location data from the GPS collars to calculate home ranges for each fox with at least 50 locations. We used the extension Home Range Tools (ver. 2.0; Centre for Northern Forest Ecosystem Research, Thunder Bay, ON, Canada) for ArcMAP (ver. 10.6; ESRI, Redlands, CA, USA). The home range for each radio-collared fox was estimated by calculating a 95% Minimum Convex Polygon (MCP). We used 95% MCPs for home ranges to avoid the inclusion of long-distance exploratory movements that would artificially inflate home range size and therefore would not be representative of the area used by foxes to satisfy life-history requirements.

For all statistical analyses, we set α at 0.10. We chose a more relaxed α value to reduce the risk of committing a Type II error, which tends to be high with small sample sizes like those in this study [22]. Detecting trends with ecological data can be challenging because all potential confounding factors cannot be controlled [23]. By reducing the Type II error rate, we were more likely to detect potential relationships that could be further investigated [24,25,26,27,28].

## 3. Results

During this study, 37 kit foxes were captured. Radio-collars were placed on 20 (10 males, 10 females) of the foxes, 16 in summer 2022 and 4 in winter 2022–2023. All 37 of the captured foxes were dye-marked to facilitate identification on field cameras. Collared kit foxes were tracked to 68 different dens over the course of 44 weeks of monitoring (24 June 2022–28 April 2023), and 390 7-day monitoring sessions were conducted at these dens.

The proportion of weeks in which another fox used a den to which a collared fox was tracked was 78.5% for the first two nights, 84.4% for the first four nights, and 89.0% for the full seven-night session. Within these intervals (Table 1), the proportion did not vary among seasons (2 nights: *χ*^2^ = 2.28, 3 df, *p* = 0.516; 4 nights: *χ*^2^ = 0.94, 3 df, *p* = 0.816; 7 nights: *χ*^2^ = 2.05, 3 df, *p* = 0.562) or between sexes (2 nights: *χ*^2^ = 1.99, 1 df, *p* = 0.158; 4 nights: *χ*^2^ = 1.28, 1 df, *p* = 0.258; 7 nights: *χ*^2^ = 0.81, 1 df, *p* = 0.368).

**Figure 3:**
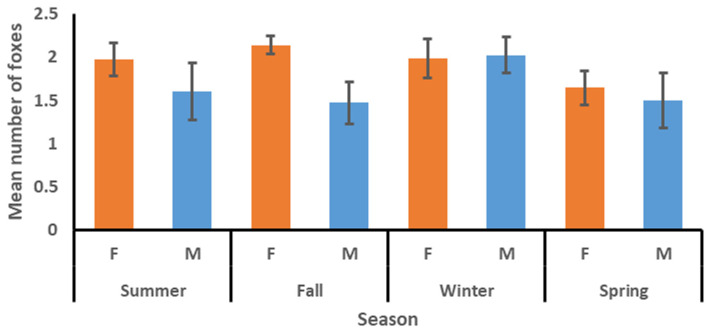
Mean number (with standard error bars) of other kit foxes using a den to which a radio-collared kit fox had been tracked in the first two nights by season and sex (females = orange bars, males = blue bars), June 2022–April 2023 in Bakersfield, CA.

**Figure 4:**
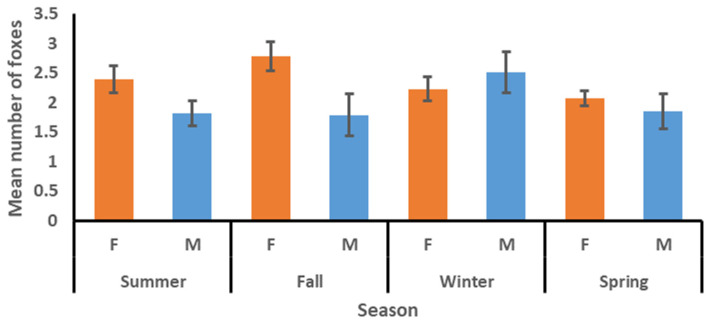
Mean number (with standard error bars) of other kit foxes using a den to which a radio-collared kit fox had been tracked in the first four nights by season and sex (females = orange bars, males = blue bars), June 2022–April 2023 in Bakersfield, CA.

**Figure 5:**
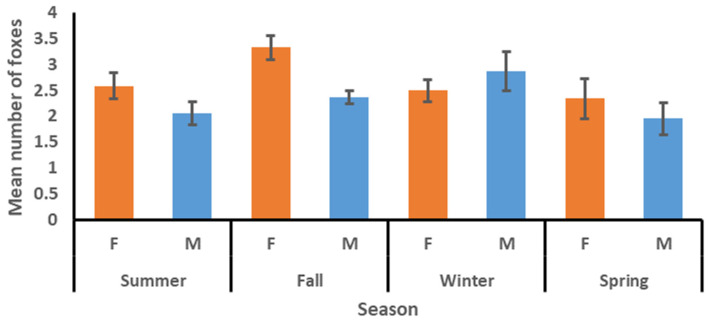
Mean number (with standard error bars) of other kit foxes using a den to which a radio-collared kit fox had been tracked in the first seven nights by season and sex (females = orange bars, males = blue bars), June 2022–April 2023 in Bakersfield, CA.

**Figure 6:**
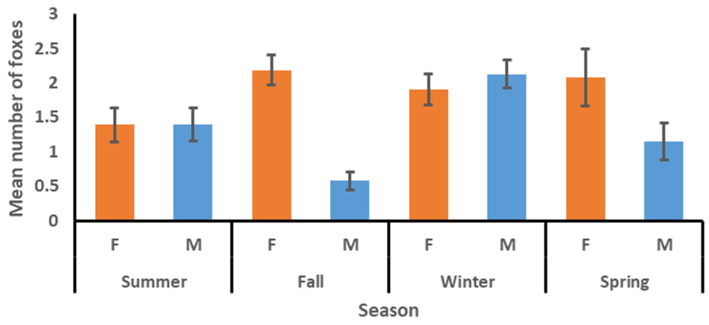
Mean number (with standard error bars) of other kit foxes documented inside a den concurrently with the tracked kit fox during a week-long monitoring session by season and sex (females = orange bars, males = blue bars), June 2022–April 2023 in Bakersfield, CA.

The mean number of other kit foxes using a den within the first two nights, four nights, and seven nights after a radio-collared fox was tracked to the den was 1.83, 2.22, and 2.52, respectively (Table 2). The mean number did not vary among factors for the two-night interval but did vary between sexes for the four-night and seven-night intervals, with the means for females being consistently higher than those for males. The mean varied among seasons for the seven-night interval and was highest in fall and lowest in spring (Table 3). The sex*season interaction was significant for the four-night and seven-night intervals, with the means for females commonly being higher than those for males in summer, fall, and spring, but lower than those for males in winter (Figure 3, Figure 4 and Figure 5). The model for the mean number of foxes found in a den concurrently with the tracked fox was significant (Table 3), with means for females being higher than those for males and means for winter and spring being higher than those for summer and fall (Table 2). The sex*season interaction was also significant, with the means for females commonly being higher than those for males in summer, fall, and spring, but lower than those for males in winter (Figure 6).

The mean number of other kit foxes using a den to which a radio-collared kit fox had been tracked was usually lowest in spring. This may have been due to foxes that reproduced, limiting the use of their dens due to the presence of young pups. Indeed, the mean number was higher for foxes not associated with pups compared to foxes associated with pups, with the differences being significant for the four-night and seven-night intervals (Table 4).

Seventeen radio-collared kit foxes were tracked for full 120-day intervals, with some of the foxes being tracked for intervals in each of the two seasons: summer–fall and winter–spring. (One fox died and two others dispersed and therefore were not tracked for a full 120-day interval.) The mean number of detections via radio-telemetry or field camera during these intervals for all foxes and seasons was 21.6 (Table 5). The mean number of dens used was 7.6 (Table 5), with no significant sex or season effects (*F*_3,27_ = 1.33, *p* = 0.286). The mean number of other foxes detected during an interval that used a den in the same week that the collared fox was detected using the den was 9.8 (Table 5), with no significant sex or season effects (*F*_3,27_ = 1.84, *p* = 0.163). The mean number of other foxes that were in the den concurrently with the collared fox was 7.3 (Table 5), with no significant sex or season effects (*F*_3,27_ = 2.21, *p* = 0.110).

**Figure 7:**
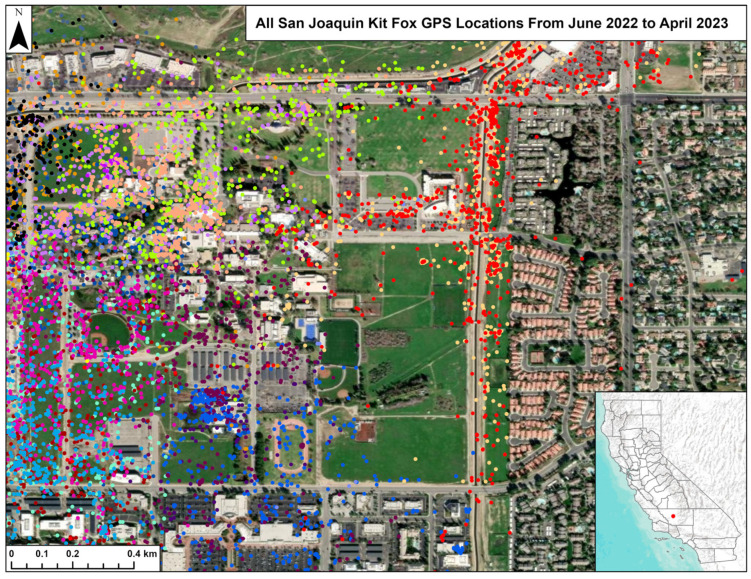
GPS locations for 19 kit foxes, June 2022–April 2023 in Bakersfield, CA. Each color represents a different fox.

**Figure 8:**
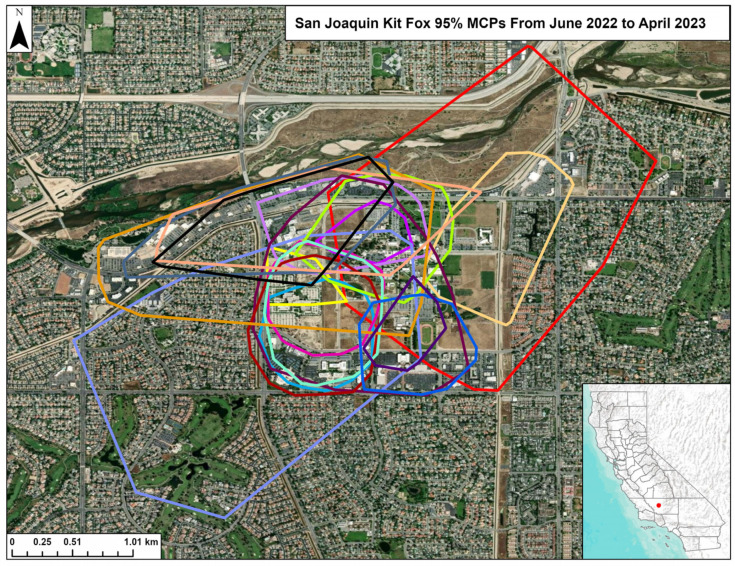
Home ranges (95% Minimum Convex Polygons) for 18 kit foxes, June 2022–April 2023 in Bakersfield, CA. One fox with a particularly large home range is not displayed. Each color represents a different fox.

One radio-collared fox died during this study, but we collected 13,945 GPS locations for the other 19 radio-collared kit foxes and used these to estimate home ranges. The locations (Figure 7) and the home ranges (Figure 8) indicated extensive spatial overlap among the foxes on the study site. We did not attempt to calculate overlap indices between home ranges given the obvious extent of overlap.

## 4. Discussion

On our study site in the urban environment of Bakersfield, we found that use of a given den by multiple kit foxes occurred quite frequently. Of significance, use of a den by other foxes commonly occurred within intervals of time during which live mange mites could potentially be present in the soil of the den following use by a fox with mange. Furthermore, two or more foxes were frequently documented in a den concurrently, indicating a high potential for direct contact and mite transmission. As many as 12 foxes were detected sharing a den concurrently with a given monitored fox during a seven-day period.

We caution that all of the estimates of den sharing should be considered conservative. For a number of reasons, den sharing was likely even higher than we observed. Occasionally, we were unable to locate a radio-collared fox, resulting in gaps in the data set for that fox of one or more weeks, during which any den sharing with other foxes was not recorded. We also occasionally observed unmarked foxes on the cameras, and it was not always clear whether we were observing a single fox or multiple unmarked foxes. When uncertain, only one fox was tallied, but the actual number could have been higher. The cameras did not always detect all of the foxes using a particular den. We base this assertion on the fact that, on occasion, radio-collared foxes were tracked to a den where a camera station was then established, but the collared fox was not detected leaving or using the den. This could have been a result of the foxes moving faster than the trigger speed of the cameras. Very commonly, a radio-collared fox was tracked to a den but was not documented using that den every day. The camera may have missed detecting the fox, as mentioned above. However, in many cases the fox could have been using another den where den sharing was not being monitored. Indeed, on numerous occasions, foxes originally tracked to one den were also detected using other dens that were also being monitored during the same week. Thus, the actual rates of den sharing were very likely higher than the rates we documented.

In general, kit fox den use patterns did not differ significantly among seasons, although den sharing trended somewhat higher in fall and winter. This may have been due to lower temperatures during these seasons and possible huddling behavior by foxes to conserve body heat. Increased den sharing during these seasons was also observed among kit foxes in natural habitats [29] and among swift foxes (*Vulpes velox*), a species closely related to kit foxes [30]. During the period of gestation and pup rearing, foxes that were parents to a litter of pups or that functioned as helpers in raising their parents’ pups shared dens less frequently than foxes that were not associated with a litter. To protect their litter, foxes likely avoid or limit interactions with the pups by foxes other than the mother, father, and any helpers. At a natural lands study site, reproducing foxes also exhibited significantly lower den sharing during February when new litters were being born [29]. The potential for mite survival and transmission within dens is likely higher in seasons such as winter and spring when temperatures are cooler and humidity is higher [17]. Although some of the kit foxes in this study did exhibit signs of mange, we did not attempt to analyze soil samples from the dens to confirm the presence of mites. Due to the depth and complexity of kit fox dens, collecting soil samples from the resting chambers, where mites are most likely to be shed, without damaging a den would be extremely difficult.

A sex bias in den sharing was apparent, with females consistently sharing more frequently than males. This trend was also observed in a natural environment with offspring from current, as well as previous, litters commonly sharing dens with their mother [29]. We were not certain of most relationships between monitored foxes and therefore could not determine whether related foxes were the ones frequently denning with adult females. Regardless, these results suggest that females may play a larger role in the spread of mange compared to males. That said, the frequency of den sharing by the males was likely sufficient to facilitate the spread of mange.

Over the course of the estimated period (120 days) during which a given kit fox could be shedding mites prior to succumbing to mange, each monitored fox used over seven dens on average, with some foxes using as many as 15 dens. During this same hypothetical period, almost 10 other foxes on average and as many as 21 foxes used the same den within one week of the den being used by the monitored fox. Finally, over seven other foxes on average and as many as 17 foxes were found using the den concurrently with the monitored fox. The observed use of multiple dens and the sharing of dens, either separately or concurrently, during this period all create abundant opportunity for a fox without mange to use a contaminated den or to come in contact with an infected fox and contract mange. Also, we emphasize that the 120-day period provides a conservative estimate of dens used and den sharing. Based on our efforts to monitor and capture and treat foxes, a number of foxes with mange lived longer than 120 days before succumbing.

**Figure 9:**
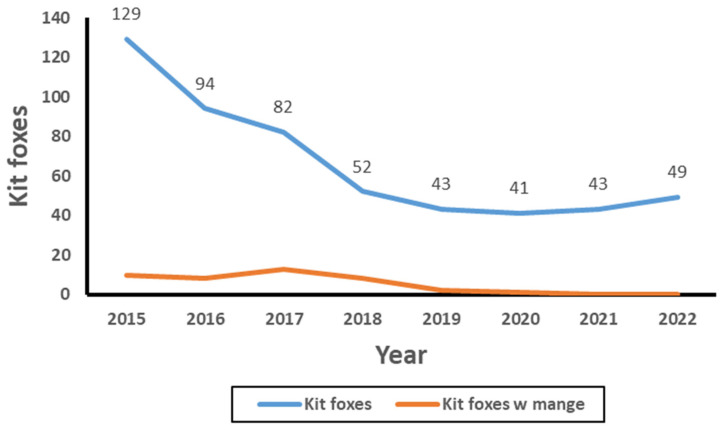
Total number of kit foxes and kit foxes with mange detected during camera station surveys in Bakersfield, CA between 2015 and 2022 [12].

Disease spread and transmission depends on the number of contacts between individuals, the probability that an infected individual will transmit the disease to a susceptible individual, and the duration of infectiousness. A significant epidemiological metric for how infectious a disease may be and how rapidly it might spread is the basic reproduction ratio known as R_0_ [31]. R_0_ is the average number of susceptible individuals that can be infected by a single diseased individual. It is a determining factor in whether an epidemic continues (R_0_ > 1) or terminates (R_0_ < 1). With regards to the kit fox population in our study, the mean number of other foxes that shared a den concurrently with a monitored fox was 7.3, which potentially results in an R_0_ much greater than 1. This high value may explain the observed rapid spread of mange among Bakersfield foxes to epidemic proportions [7,32] that eventually resulted in an apparent population decline (Figure 9).

Mange has not been detected among kit foxes in natural habitats, even those adjacent to the Bakersfield urban environment [33]. Foxes, including some with mange, routinely cross the interface between urban and natural lands [33]. Kit foxes in natural habitats commonly use multiple dens during the course of a year with mean (range) estimates per fox including 8.4 (1–31) [34], 11.8 (1–16) [29], 13.0 (3–23) [35], 15.6 (9–25) [36], 16.0 (1–58) [37], 17.6 (1–64) [38], and 19.4 [39]. Den sharing also commonly occurs among kit foxes in natural habitats [29,40]. This all suggests that the potential for mange to spread into and throughout kit fox populations in natural habitats should be high.

The social ecology of kit foxes may offer some explanation for the apparent absence of mange in natural populations. Foxes that share dens are almost always related [13,29,40]. Den sharing between individuals from different social groups is rare and apparently primarily occurs during pair formation when a male and a female from different social groups attempt to form a pair [13]. Otherwise, foxes from different social groups do not share dens. Even if foxes from adjacent family groups used a common den along the margin of their ranges, concurrent use is unlikely, and an uninfected fox would need to use the den within a week of it being used by a fox with mange in order for the disease to spread between the groups.

In urban kit fox populations, high survival, high reproductive success, abundant resources, and fewer vacant home ranges for dispersing foxes to move into result in higher fox densities compared to populations in natural habitats [5,18]. This results in extensive spatial overlap, as was documented among the home range polygons of the monitored foxes on our study site (see Figure 7 and Figure 8). This overlap likely results in even greater den sharing, including among social groups, even if the shared dens are not used concurrently by members of different groups. Extensive spatial overlap and den sharing was also observed in a high-density population of bare-nosed wombats, a species also impacted by mange [29]. Similarly, mange would be expected to spread rapidly among any other mammalian species that routinely use earthen dens and where use of such dens by multiple conspecifics is common.

## 5. Conclusions

The patterns of den use by kit foxes observed in this study and the implications for mite transmission indicate that preventing the spread of sarcoptic mange in the urban population will be quite challenging. One possibility we had considered was some form of den treatment, similar to the strategy used to treat prairie dog (*Cynomys* spp.) burrows to kill the fleas that transmit plague [41,42]. However, just locating the multiple dens used by foxes would be difficult, and treating the den would not necessarily kill the mites on the foxes. Also, unless some sort of long-acting treatment that was safe for the kit foxes was available, a treated den could immediately be recontaminated by a fox with mange.

The den use patterns also highlighted the number of kit foxes that could potentially be infected by a single fox. Clearly, if a fox is detected with mange in a given area, the probability is high that a number of other foxes in the same area also are infected. This is consistent with our experiences in trapping to treat foxes with mange. In most instances, we have captured other foxes that also have mange. Consequently, it is important to continue trapping, even once the fox that was originally detected has been captured and treated, until no new animals are captured.

## Figures and Tables

**Table 1:** Proportion of monitoring sessions that another kit fox used a den to which a radio-collared kit fox had been tracked in the first two nights, first four nights, and all seven nights of a monitoring session, June 2022–April 2023 in Bakersfield, CA.

		Proportion of Sessions (%)		
	n	2 Nights	4 Nights	7 Nights
Total	390	78.5	84.4	89.0
Season:				
Summer	99	73.7	81.8	85.9
Fall	68	82.4	83.8	92.6
Winter	104	77.9	84.6	88.5
Spring	119	80.7	86.6	89.9
Sex:				
Female	264	80.7	86.0	90.2
Male	126	73.8	81.0	86.5

**Table 2:** Mean number of other kit foxes using a den to which a radio-collared kit fox had been tracked in the first two nights, first four nights, and all seven nights of a monitoring session, and the mean number of other foxes during the session documented inside the den concurrently with the tracked fox, June 2022–April 2023 in Bakersfield, CA. In each column, seasonal means with the same letter were not significantly different.

		2 Nights		4 Nights		7 Nights		Foxes in Den Concurrently	
	n	Mean (SE)	Max	Mean (SE)	Max	Mean (SE)	Max	Mean (SE)	Max
Total	390	1.83 (0.07)	8	2.22 (0.08)	9	2.52 (0.09)	12	1.77 (0.09)	12
Season:									
Summer	99	1.80 A (0.16)	6	2.11 A (0.16)	6	2.33 BC (0.17)	6	1.39 B (0.17)	6
Fall	68	1.96 A (0.17)	6	2.50 A (0.21)	6	3.06 A (0.20)	7	1.74 B (0.21)	5
Winter	104	2.00 A (0.17)	8	2.34 A (0.19)	9	2.63 AB (0.21)	12	1.99 A (0.21)	12
Spring	119	1.62 A (0.10)	4	2.03 A (0.11)	4	2.28 C (0.11)	4	1.92 AB (0.12)	4
Sex:									
Female	264	1.89 (0.08)	8	2.30 (0.09)	9	2.61 (0.10)	12	1.92 (0.10)	10
Male	126	1.70 (0.14)	8	2.03 (0.15)	9	2.34 (0.17)	12	1.46 (0.17)	12

**Table 3:** Results of two-way analysis of variance for the mean number of other kit foxes using a den to which a radio-collared kit fox had been tracked in the first two nights, first four nights, and all seven nights of a monitoring session, and the mean number of other foxes during the session documented inside the den concurrently with the tracked fox, June 2022–April 2023 in Bakersfield, CA. *p*-values in bold were considered significant at α = 0.1.

	F	df	*p*
2-night interval:			
Model	1.33	7382	0.233
Sex	2.97	1382	**0.085**
Season	1.20	3382	0.310
Sex*Season	0.81	3382	0.489
4-night interval:			
Model	2.08	7382	**0.045**
Sex	4.21	1382	**0.041**
Season	1.06	3382	0.367
Sex*Season	2.23	3382	**0.085**
7-night interval:			
Model	2.95	7382	**0.005**
Sex	3.79	1382	**0.052**
Season	2.55	3382	**0.055**
Sex*Season	2.25	3382	**0.083**
Foxes in den concurrently:			
Model	3.72	7382	**<0.001**
Sex	8.91	1382	**0.003**
Season	2.81	3382	**0.039**
Sex*Season	4.53	3382	**0.004**

**Table 4:** Comparison of the mean number of other kit foxes using a den to which a radio-collared kit fox had been tracked in the first two nights, first four nights, and all seven nights of a monitoring session for reproducing and non-reproducing foxes in spring 2023 in Bakersfield, CA.

	Mean (SE)		
	Reproducing (n = 102)	Non-Reproducing (n = 17)	*t*_1,117_ *p*
2-night interval	1.56 (0.10)	2.0 (0.26)	2.57 0.112
4-night interval	1.94 (0.12)	2.59 (0.29)	4.31 0.040
7-night interval	2.19 (0.12)	2.82 (0.29)	4.07 0.046

**Table 5:** Season and sex comparison of the mean number of detections, dens used by radio-collared kit foxes, other kit foxes using a den to which a radio-collared kit fox had been tracked, and other kit foxes using a den concurrently with a radio-collared fox within 120 days of a radio-collared fox being tracked to a den between June 2022 and April 2023 in Bakersfield, CA.

	Season		Sex		
	Summer–Fall	Winter–Spring	Female	Male	Total
n	16	15	18	13	31
Detections					
Mean (SE)	13.3 (1.5)	30.5 (2.7)	23.3 (2.4)	19.3 (4.0)	21.6 (2.2)
Range	6–30	8–59	8–36	6–59	6–59
Dens used					
Mean (SE)	6.7 (0.5)	8.5 (1.0)	7.5 (0.6)	7.6 (1.0)	7.6 (0.5)
Range	4–10	2–15	3–11	2–15	2–15
Other foxes same week					
Mean (SE)	9.4 (0.9)	10.2 (1.2)	9.5 (0.9)	10.2 (1.3)	9.8 (0.8)
Range	4–16	4–21	4–16	4–21	4–21
Other foxes concurrently					
Mean (SE)	7.1 (0.8)	7.5 (1.0)	6.9 (0.7)	7.8 (1.2)	7.3 (0.6)
Range	2–14	2–17	2–14	2–17	2–17

## Data Availability

Data will be made available upon reasonable request.
